# A high-content imaging assay for the quantification of the *Burkholderia pseudomallei* induced multinucleated giant cell (MNGC) phenotype in murine macrophages

**DOI:** 10.1186/1471-2180-14-98

**Published:** 2014-04-22

**Authors:** Gianluca Pegoraro, Brett P Eaton, Ricky L Ulrich, Douglas J Lane, Jenifer F Ojeda, Sina Bavari, David DeShazer, Rekha G Panchal

**Affiliations:** 1 Molecular and Translational Sciences Division, United States Army Medical Research Institute of Infectious Diseases, 1425 Porter Street, Fort Detrick, Frederick, MD 21702-5011, USA; 2 Perkin Elmer, Waltham, MA 02451, USA; 3 Present Address: Center for Cancer Research, National Cancer Institute/NIH, Bethesda, MD 20892, USA

**Keywords:** *Burkholderia pseudomallei*, High Content imaging, phagocytosis, Multinucleated giant cells, Macrophages

## Abstract

**Background:**

*Burkholderia pseudomallei* (Bp), a Gram-negative, motile, facultative intracellular bacterium is the causative agent of melioidosis in humans and animals. The Bp genome encodes a repertoire of virulence factors, including the cluster 3 type III secretion system (T3SS-3), the cluster 1 type VI secretion system (T6SS-1), and the intracellular motility protein BimA, that enable the pathogen to invade both phagocytic and non-phagocytic cells. A unique hallmark of Bp infection both *in vitro* and *in vivo* is its ability to induce cell-to-cell fusion of macrophages to form multinucleated giant cells (MNGCs), which to date are semi-quantitatively reported following visual inspection.

**Results:**

In this study we report the development of an automated high-content image acquisition and analysis assay to quantitate the Bp induced MNGC phenotype. Validation of the assay was performed using T6SS-1 (∆*hcp1*) and T3SS-3 (∆*bsaZ*) mutants of Bp that have been previously reported to exhibit defects in their ability to induce MNGCs. Finally, screening of a focused small molecule library identified several Histone Deacetylase (HDAC) inhibitors that inhibited Bp-induced MNGC formation of macrophages.

**Conclusions:**

We have successfully developed an automated HCI assay to quantitate MNGCs induced by Bp in macrophages. This assay was then used to characterize the phenotype of the Bp mutants for their ability to induce MNGC formation and identify small molecules that interfere with this process. Successful application of chemical genetics and functional reverse genetics siRNA approaches in the MNGC assay will help gain a better understanding of the molecular targets and cellular mechanisms responsible for the MNGC phenotype induced by Bp, by other bacteria such as *Mycobacterium tuberculosis*, or by exogenously added cytokines.

## Background

*Burkholderia pseudomallei* (Bp) is a Gram-negative bacterial pathogen and the causative agent of melioidosis, a potentially fatal disease if misdiagnosed or left untreated [1,2]. Bp is endemic to Southeast Asia, Northern Australia, South America, Africa, Middle East, China and India and the pathogen can be commonly isolated from soil and surface waters [1,3,4]. Both acute and chronic infections with Bp can be acquired by inhalation, percutaneous inoculation and in rare circumstances by ingestion. The clinical symptoms of melioidosis are broad and may present as acute or chronic pneumonia, internal organ abscesses (lung, liver and spleen), fulminating septicemia and uncommonly individuals can be asymptomatic [1]. In fact, and due to the facultative intracellular lifestyle of Bp, dormant cases have been reported with the most notable being 62 years after initial exposure [5]. With the relative ease of genetic manipulation, environmental availability and intrinsic antibiotic resistance, Bp is listed as a category B select agent by the U.S. Centers for Disease Control and Prevention [6].

Macrophages and monocytes play critical roles in both the innate and adaptive arms of the immune system and are the first line of host defense mediating immunological responses to foreign antigens [7,8]. These cells have diverse functions within the host including phagocytosis of bacterial, fungal, parasitic and viral pathogens, cytokine and chemokine biosynthesis for inflammatory mediated responses to invading pathogens as well as regulation of cellular metabolic processes including fatty acid metabolism, iron reprocessing and mineral reabsorption [9-11]. In response to certain biological triggers, monocytes or macrophages form multinucleated giant cells (MNGCs), which involves the fusion of adjacent cells and results in a multinucleated cell with a single cytoplasmic compartment [12]. MNGCs are a well characterized phenotype in tissue granuloma formation in response to bacterial infection, with the most notable being associated with *Mycobacterium tuberculosis* (Mtb). Using various animal, human, *in vitro* cell culture and explant tissue models of Mtb infection it has been demonstrated that monocytes develop into various MNGC types, which is essential in the confinement of Mtb within infectious granulomas [13-20]. Likewise, monocyte and macrophage MNGC formation can be induced *in vitro* using various conditioned mediums containing exogenous cytokines, lectin, phorbol myristate acetate and even select antibodies [21-32]. The most notable cytokines associated with monocyte and macrophage differentiation into MNGCs are Interleukin-4 (IL-4) and Interferon gamma (IFN-γ). However, recent reports have also demonstrated that MNGC formation is dependent on diverse range of cellular proteins including CD36, TREM-2, E-cadherin, CCL2 and Rac1, MMP9, DC-STAMP, E-cadherin and Syk; all of which are involved in intracellular signaling, cell surface communication, proteolysis, chemotaxis and cellular transcription [28,33-43].

A unique phenotypic characteristic of Bp infection, in addition to *Burkholderia mallei* (Bm) and *Burkholderia thailandensis* (Bt), is the ability to induce host cell MNGC formation following cellular uptake, in both tissue culture cells (i.e. murine macrophages) and in primary human cells (patients with active melioidosis) [44-47]. MNGC formation has been demonstrated in both phagocytic and non-phagocytic cells in addition to patient tissue(s) with active melioidosis [46-54]. The importance of Bp-mediated MNGC formation during infection is currently unknown, but it is possible that cell to cell spread via MNGC allows the pathogen to avoid immune surveillance *in vivo*. The Bp genome encodes a diverse range of specialized protein secretion systems including three type III secretion systems (T3SS) and six type VI secretion systems (T6SS) [1,55,56]. Mutation of the Bp T3SS-3, which is homologous to the *Shigella* Mxi-Spa and *Salmonella* SPI-1 T3SSs, results in loss of Bp induced MNGC formation, inability of endosomal escape and loss of virulence in animal models of Bp infection [50,53,57]. Likewise, disruption of components making up the T6SS-1 reduced animal virulence and hindered MNGC formation in RAW264 macrophages [58]. In addition, it has been shown that the Bp alternative sigma factor RpoS, which is involved in genome-wide regulation of bacterial adaptation to environmental stress (i.e. nutrient limitation), plays a role in Bp induced MNGC formation [59]. Recently, the molecular mechanism of Bp MNGC formation was revealed by Toesca *et al.*[60]. The T6SS-1 valine-glycine repeat tail spike protein (VgrG1) possesses a novel fusogenic domain at its C-terminus that mediates cell fusion and allows Bp cell to cell spread.

Automated high content imaging (HCI) microscopy is a powerful technique to quantitatively characterize cellular phenotypes at the single cell level in response to bacterial and viral infection, exposure to drug agonists and antagonists and for drug mechanism of action determination [61-69]. This work describes the development of a cell-based HCI immunofluorescence assay to quantitatively characterize the MNGC phenotype induced in murine macrophages upon infection with Bp K96243. As a proof of principle for its applicability in a relevant biological setting, this assay was validated using mutants of Bp that were previously described to be defective for MNGC formation in mouse macrophages [58,70]. Furthermore, we used the MNGC HCI assay to screen a focused small molecule library to identify compounds that interfere with MNGC formation induced by Bp. Together, the results of these experiments indicated that the HCI MNGC assay can be used in a medium-throughput format to identify and characterize Bp mutants that are defective in their ability to induce MNGCs and to identify small molecules that inhibit this phenotype.

## Results & discussion

### Optimization of the MNGC assay

**Figure 1:**
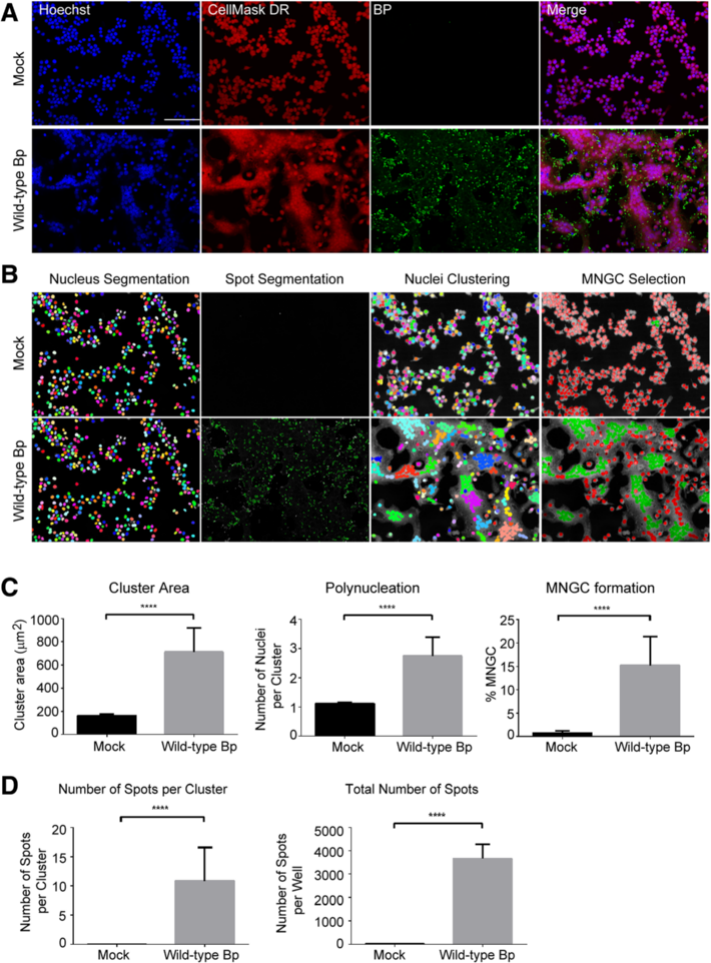
**Quantitative analysis of *B. pseudomallei *K96243 induced murine macrophage MNGC formation. (A)** Representative 20X magnification confocal images of RAW264.7 macrophages that were not infected (mock) or infected with wild-type *B. pseudomallei* K96243 at a MOI of 30 at 10 h post-infection. Images of cell nuclei (stained with the Hoechst 33342 dye), cell cytoplasm (stained with CellMaskDR- CellMask DeepRed) and Bp: bacteria labeled using an anti-*B. pseudomallei* mouse monoclonal and a secondary anti-mouse/Alexa488 antibody. Scale bar: 90 μm. **(B)** Visual representation of the MNGC Image Analysis procedure. Each object (Nuclei) is pseudocolored with a unique color in the nucleus segmentation panel. Bacterial spots are pseudocolored in green in the spot segmentation panel. Nuclei clustering: Nuclei are clustered based on distance as described in Experimental procedures to generate the Cluster population. In the MNGC selection panel, image objects classified as MNGC are pseudocolored in green, and non-MNGC objects are pseudocolored in red. **(C)** Histograms representing the quantification of cellular attributes of the cluster population as measured by the MNGC image analysis procedure described in Figure 1B. **(D)** Histograms showing the results of the quantification of cellular attributes related to bacterial spot formation. In **C** and **D** means +/- standard deviation (SD) are shown for three independent *B. pseudomallei* macrophage infections performed on separate days and with six replicates/plate. n = 18 and > 500 nuclei were analyzed per well. **** p < 0.0001.

To develop an automated high-throughput method for quantitating MNGCs, RAW264.7 macrophages were either not infected (Figure 1A, Top panel-mock) or infected at an MOI of 30 with wild-type Bp K96243 (Figure 1A, bottom panel-wild-type Bp). After 2 h excess extracellular bacteria were then eliminated by sequential washes in PBS and cells were further incubated in medium containing kanamycin. At 10 h post-infection macrophages were first fixed, and then immunofluorescence (IF) staining was performed to detect bacteria and cellular structures. Finally, samples were imaged by high-throughput confocal fluorescence microscopy. Cell nuclei were stained with the DNA dye Hoechst 33342 and the cell body with the CellMask DeepRed dye. Bacteria associated with or internalized by macrophages were detected by staining cells with an anti-*Burkholderia pseudomallei* monoclonal antibody.

As observed in the fluorescence microscopy images, Bp infection induced cell-to-cell fusion, clustering of the nuclei and cell body enlargement in a substantial fraction of infected macrophages when compared to mock infected samples (Figure 1A). These cellular objects fit the definition of MNGC. A large number of Bp bacterial spots were found to be either internalized or in close proximity with the boundaries of infected cell bodies. In these experimental conditions not all the infected cells appear to be part of an MNGC object (Figure 1A). Hence, it was important to develop an HCI analysis that would recognize and distinguish MNGC objects from non-MNGC objects in a heterogeneous population of infected cells. To address this issue, we took advantage of the close proximity of the nuclei in MNGC’s to recognize and classify MNGC clusters. Briefly, and as shown in Figure 1B, cell nuclei were first identified by using the Hoechst 33342 channel image, thus obtaining a population of objects that was named “Nuclei”. The cell body edges were identified by expanding the body of the nucleus detected in the previous step. The cell body borders were then detected by using the CellMask DeepRed channel image.

Bp spots were identified using the Bp antibody channel image. Several cellular attributes were calculated for the Nuclei population, the most relevant being: number of objects, cell body area and number of bacterial spots per object. The next step in the image analysis consisted in recursively clustering distinct Nuclei objects together into a single “Cluster” object, provided that their nuclei were either touching or adjacent. All the cellular attributes of the Nuclei population calculated on a single-object basis were then summed into the corresponding Cluster object. In addition, the number of Nuclei per Cluster (Polynucleation) was calculated. Finally, based on visual inspection of images analyzed with this strategy, the Cluster population was further classified into either MNGC (>3 Nuclei per Cluster) or non-MNGC (≤3 Nuclei per Cluster) sub-populations (Figure 1B). This approach was then used to quantitatively measure MNGC formation in RAW264.7 macrophages infected with wild-type Bp K96243. As seen in Figure 1C, the results of these experiments indicate that the HCI MNGC analysis can be used at the well level to detect MNGC formation in Bp K96243-infected populations when compared to mock infected samples. In particular, and as expected, infected cells had a 4.3-fold increase in Cluster Area, a 2.4-fold increase in Number of Nuclei per Cluster, and a 21-fold increase in the Percentage of MNGC when compared to non-infected samples.

### Single cell analysis of the Bp K96243 infected macrophages

**Figure 2:**
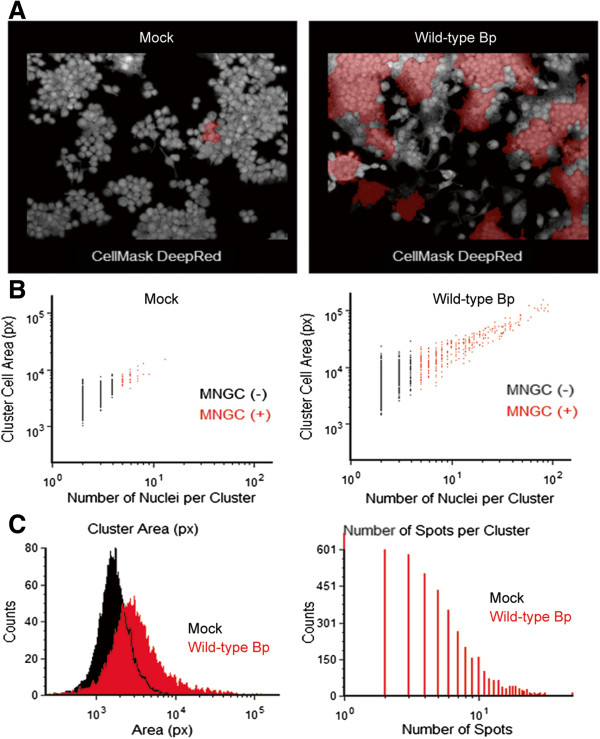
**Single cell analysis of *B. pseudomallei *K96243 induced murine macrophage MNGC formation. (A)** Representative 20X magnification confocal images of RAW264.7 macrophages that were not infected (Mock) or infected with wild-type *B. pseudomallei* K96243 at a MOI of 30 at 10 h post-infection. CellMask DeepRed –cytoplasmic/nuclear stain. **(B)** Single cell image cytometry analysis of MNGCs induced in macrophages that were not infected (Mock; left panel) or infected with wild-type *B. pseudomallei* K96234 (right panel). Objects classified as MNGC (+) are pseudocolored in red in the image plots and in the dot plot graphs. **(C)** Histogram plots showing the distribution of the cluster populations based on the cluster area (left panel) in macrophages that were uninfected (Mock, black) or infected with wild-type *B. pseudomallei* K96234 (Wild-type Bp, red); and the number of bacterial spots associated with each cluster (right panel).

Quantitation of MNGCs using the image analysis procedure typically outputs statistical descriptors, such as means and standard deviations, at the well level. While the well level analysis of MNGC formation provides statistically significant differences between mock infected and Bp K96243 infected cells (Figure 1B), we also wanted to determine if our image analysis approach was capable of distinguishing MNGCs in heterogeneous populations of infected cells. To test this, we plotted single-cell data generated by the MNGC analysis on either mock-infected or Bp K96243 infected cells (Figure 2). As expected, using a similar classification approach to the one described above, we were able to visually detect an increase in the incidence of MNGC formation in images from Bp K96243 infected macrophages compared to uninfected macrophages (Figure 2A). The percentage of Cluster objects classified as MNGC (+) increased from 0.52% (mock) to 6.6% (Bp K96243) (Figure 2B). The presence of a small percentage of MNGC (+) objects in uninfected RAW264.7 samples reflects the presence of cell clumps morphologically unrelated to real MNGC (Figure 2A and Figure 2B) and constitutes the negative control measurement background in the MNGC analysis. Nevertheless, as expected, clusters classified as MNGC (+) in Bp K96243 infected samples had larger mean Cluster Area and a larger mean Number of Spots per Cluster when compared to the MNGC (-) objects present in the same samples at the 10 h time point. Accordingly, the higher incidence of MNGC (+) objects in Bp K96243 infected cells when compared to mock infected cells led to a shift towards higher values of Cluster Area and Number of Spots per Cluster in the single-cell distributions (Figure 2C). Thus, the results of the MNGC HCI analysis indicate that, at an MOI of 30 and 10 h post Bp K96243 infections, there are at least two sub-populations of RAW264.7 cells associated with bacterial spots, which are phenotypically dissimilar, both in terms of cell size and polynucleation (Figure 2B and Figure 2C).

### Validation of the MNGC assay to detect mutants defective in their ability to induce MNGC

**Figure 3:**
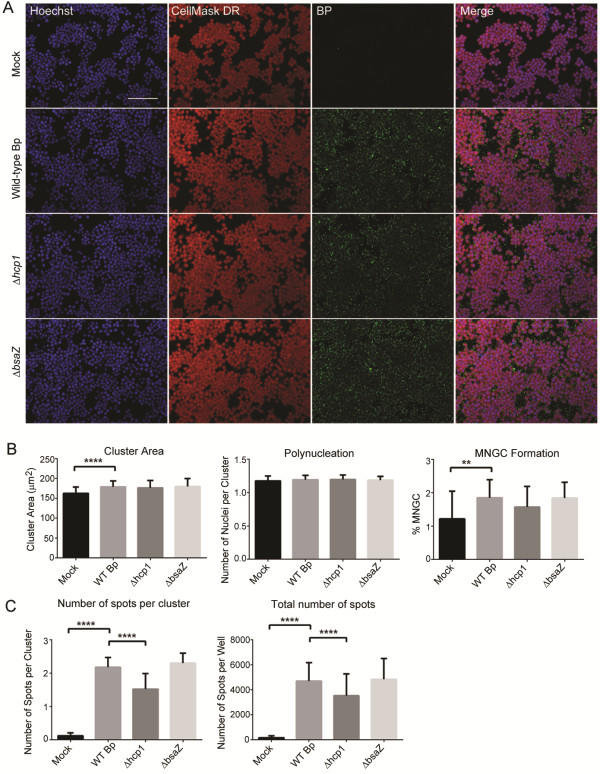
**Validation of the MNGC assay (2 h post**-**infection). (A)** Representative confocal images of RAW264.7 macrophages infected at 30 MOI with wild-type Bp K96243 (wt), or Bp ∆*hcp1*, or Bp ∆*bsaZ* respectively. Scale bar: 90 μm. Macrophages were infected with Bp for 2 h and then fixed, processed in IF and images were acquired and analyzed according to the MNGC analysis script (described in the Methods - Image acquisition and analysis section and shown in Figure 1B). **(B)** Bar graphs for the quantification of several cellular features of MNGC formation. **(C)** Bar graphs for the quantification of bacterial spots per MNGC cluster and total number of bacterial spots. In **B** and **C** means +/- SD are shown of 6 replicates per plate, 3 plates run on independent days (n = 18). For each replicate well >1000 nuclei were analyzed. **** p <0.0001; ** p < 0.01.

Having shown that the HCI MNGC assay is capable of detecting and quantitating Bp induced cell-to-cell fusion, we then set out to test whether this method could be used to detect defects in MNGC formation caused by mutations in Bp genes. It was previously reported that deletion of the Bp ∆*hcp1* gene, which is encoded within the cluster 1 type VI secretion system operon, resulted in a significant increase in the 50% lethal dose in a Syrian hamster model of infection (10^3^ vs. <10 bacteria), in reduced macrophage intracellular replication and most notably in the failure to induce macrophage MNGC formation [58]. Likewise, it was demonstrated that deletion or inactivation of the Bp *bsaZ* gene, which is encoded within the Bp T3SS-3 results in delayed macrophage vacuolar escape, in reduced intracellular replication at 3, 6, and 12 h and in sporadic MNGC formation [50]. Thus, in order to test the possibility of using the HCI MNGC assay to profile Bp mutants, we analyzed the ability of Bp K96243 and the two isogenic mutants harboring gene deletions in the Bp T6SS-1 (∆*hcp1*) and the T3SS-3 (∆*bsaZ*) to induce MNGC formation at two different time points. RAW264.7 macrophages were not infected (mock), infected with wild-type Bp K96243 or with the ∆*hcp1* or ∆*bsaZ* mutants at a MOI of 30 for 2 h and then processed in IF and HCI as described above (Figure 3). At the early time point (2 h), infection with all the three Bp strains led to the appearance of bacterial foci either in the cytoplasm or associated with the cell membrane of RAW264.7 macrophages (Figure 3A). When quantified with the MNGC analysis pipeline we could detect significant differences between the Bp K96243 (wt) and the mock infected samples in terms of mean Number of Spots per Clusters, Cluster Area and marginally significant differences in terms of mean Percentage of MNGC (Figure 3B). Bp ∆*hcp1* had a significantly lower Number of Spots per Clusters and a significantly lower total Number of Spots when compared to Bp K96243 (wt) (Figure 3C). At this time point, Bp ∆*bsaZ* was indistinguishable from Bp K96243 (wt) (Figure 3C). Altogether the results of these experiments indicate that deletion of *bsaZ* has no effect on bacterial adhesion and/or uptake by RAW264.7 cells, while deletion of ∆*hcp1* has some minor but significant effects on these processes. Our observed results for the Bp ∆*bsaZ* mutant were similar to that reported by French *et al*. [44]. On the contrary, our findings with Bp ∆*hcp1* mutant during this early infection time did not correlate with those reported [44,58], which may due to the differences in the experimental conditions such as MOI, time of infection or the type of *Burkholderia* strain used in the studies.

**Figure 4:**
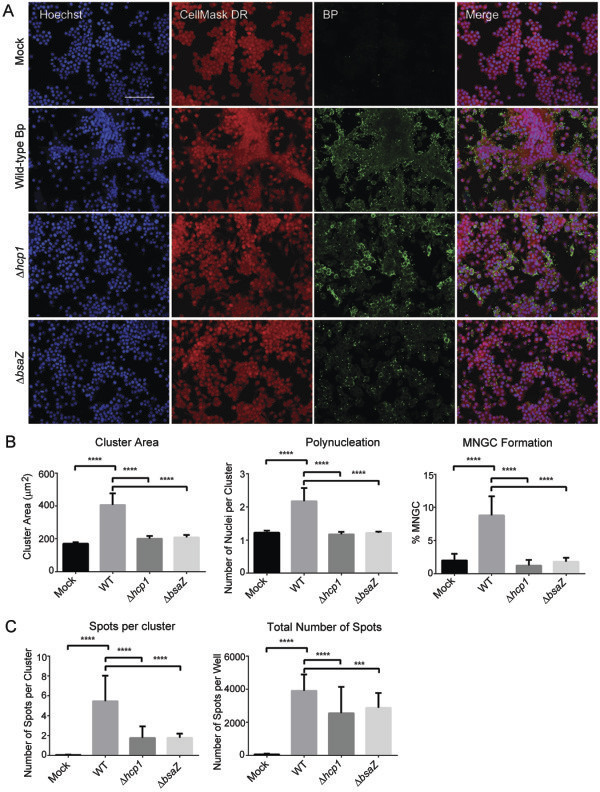
**Validation of the MNGC assay (10 h post**-**infection). (A)** Same as Figure 3A, except that macrophages were fixed at 10 h post-infection for different strains of Bp. Scale bar: 90 μm. **(B)** HCI quantification of several cellular features of MNGC formation and **(C)** bacterial features from images acquired as described in Figure 3A. In **B** and **C** means +/- SD are shown of 6 replicates per plate, 3 plates run on independent days (n = 18). For each replicate well >1000 nuclei were analyzed. **** p <0.0001; *** p < 0.001.

At later stages of the bacterial replication cycle (10 h post-infection), more significant differences were observed between Bp K96243 (wt) and the mutant strains (Figure 4). Of note, the bacterial mutants showed more diffused (∆*hcp1*) or rounder, reduced and more isolated spot staining pattern (∆*bsaZ*) when compared to Bp K96243 (wt) (Figure 4A, Bp panels). As expected, Bp K96243 (wt) infection strongly induced MNGC formation, while in this respect both Bp ∆*bsaZ* and Bp ∆*hcp1* were defective (Figure 4A, Hoechst and CellMask DR panels). HCI analysis was used to quantify differences between Bp K96243 (wt) and the bacterial mutant strains in their potential to induce the MNGC phenotype in infected RAW264.7 macrophages (Figure 4B and Figure 4C). In these experimental conditions Bp K96243 (wt) induced a 2-fold increase in mean Cluster Area and mean Number of Nuclei per Cluster and a 4-fold increase in mean Percentage of MNGC when compared to the negative control (Figure 4B). All these differences were statistically significant. On the contrary, using the same experimental conditions, while bacterial spots were still detected in IF (Mean Total Number of Spots per well, Figure 4C), Bp ∆*hcp1* or Bp ∆bsaZ failed to induce an increase in the mean Cluster Area, Number of Nuclei per Cluster or Percentage MNGC and were hardly distinguishable from the uninfected samples (Figure 4B). Significant differences in the mean number of Spots per Cluster between Bp K96243 (wt) and Bp ∆*hcp1* or Bp ∆*bsaZ* were observed (Figure 4C) and were probably due at least in part to an increase in the mean Cluster Area in Bp K96243 infected samples (see above). The inability to see an increase in the total number of bacterial spots during the intracellular replication step (10 h post-infection) compared to early uptake or phagocytosis step (2 h post-infection) may partly be due to the killing of the internalized bacteria by the professional phagocytes. Although bacteria can be detected and quantitated by HCI, this technique it does not measure bacterial viability. Altogether, these results show that the HCI MNGC assay can be implemented to quantitatively characterize mutant Bp strains phenotype based on cellular morphological changes induced in infected host cells. Furthermore, our HCI results regarding reduced MNGCs and bacterial spots following infection with Bp ∆*hcp1* or Bp ∆*bsaZ* mutants compared to wild type Bp at 10 h post-infection are consistent with previously published data [44,58].

### Screening of a small molecule library in the MNGC assay

**Figure 5:**
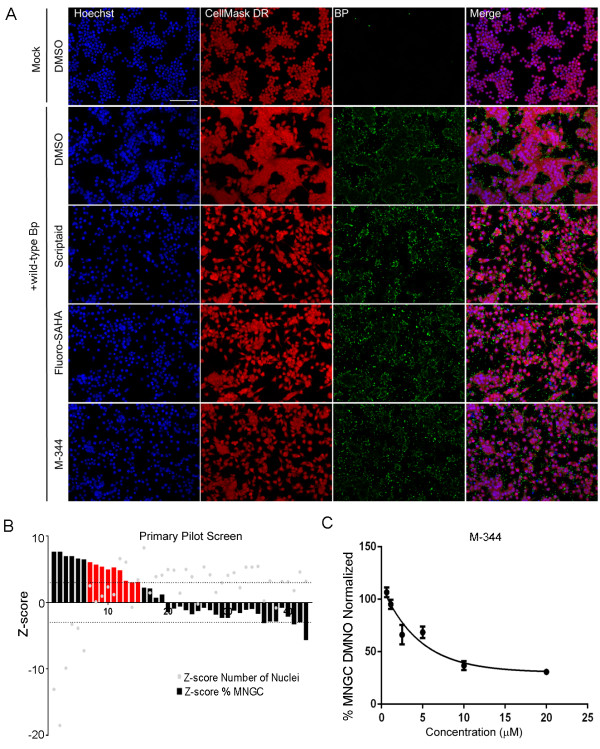
**Screening of focused small molecule library for inhibitors of MNGC formation.** RAW264.7 macrophages were pretreated for 2 h with a collection of 43 compounds active against enzymes involved in epigenetics regulation at a concentration of 20 μM and then infected with 30 MOI of Bp K96243 for 8 h. Cells were fixed, stained in IF and imaged as described above. The effect of the tested compounds on MNGC formation was quantified. Compounds were ranked based on the potency of MNGC inhibition when compared to DMSO-treated, Bp K96243-infected samples (Negative control). Cytotoxic (Number of Nuclei Z-score < -3) were not further considered. **(A)** Representative confocal images of macrophages pre-treated with DMSO control or primary hit compounds active in the MNGC screen. Scale bar: 90 μm. **(B)** Compounds that significantly reduced the number of MNGC when compared to DMSO treated samples (% MNGC Z-score > 3) were scored as positive hits (red bars). Bars represent means from two replicates. **(C)** Dose-dependent inhibition of MNGC formation by compound M-344 identified in the primary screen.

To discover possible cellular pathways that are hijacked by Bp and that might regulate cell-to-cell fusion, we used the HCI MNGC assay to screen a small, functionally focused collection of 43 compounds in duplicate. The compounds in this collection are annotated as targeting pathways involved in the epigenetic regulation of chromatin (See Experimental procedures for details). Bacterial infection induced epigenetic changes such as histone modifications, DNA methylation, chromatin remodeling, which in turn affect host cell signaling has been shown to either promote host defense or increase susceptibility to infection [71]. To investigate Bp induced epigenetic changes which in turn may modulate MNGC formation, RAW264.7 macrophages were first pre-treated with the compound library and then infected with Bp K96243. Cells treated with DMSO (Vehicle) and infected with Bp K96243 were considered as negative controls. At 8 h post-infection cells were fixed and processed in IF for the HCI MNGC assay as described above. Representative images of macrophages that were not infected (mock) or infected with Bp K96243 in presence of DMSO or identified hit compounds are shown in Figure 5A. Compounds were ranked based on the Z-score for the Percentage of MNGC attribute (Figure 5B). Compounds with high Z-scores were inhibitors of Bp K96243 induced MNGC formation, whereas compounds with low Z-scores increased MNGC formation. Compounds that had a percentage of MNGC Z-score >3 were scored as positive hits. A total of 15 out of the original 43 compounds matched this criterion (Figure 5B). Furthermore, to exclude cytotoxicity as the leading mechanism of action for MNGC reduction, compounds that had a Number of Nuclei Z-score < - 3 were not considered for further analysis. A total of 9 out of the original 15 compounds passed the cytotoxicity filter (Figure 5B) and were considered as hits. A total of 7 out of the 9 identified hits belong to the Histone Deacetylase (HDAC) enzyme inhibitor category. Importantly, none of these hit compounds reduced the total number of Bp spots per well (Data not shown), ruling out that their mechanism of action involves direct inhibition of bacterial adhesion and/or uptake by host cells. Visual inspection of samples treated with the three HDAC inhibitors (Scriptaid, Fluoro-SAHA, and M-344) confirmed that these compounds were not cytotoxic and hence did not alter the cell number when compared to DMSO treated samples, but substantially inhibited MNGC formation in their presence. Furthermore, M-344 showed a dose-dependent inhibition of MNGC formation induced upon Bp K96243 infection (Figure 5C). Altogether, these results indicate that the HCI MNGC assay can be used to screen small molecule libraries for the identification of compounds that can inhibit MNGC formation and that one or more HDAC’s might be involved in the positive regulation of this process.

## Conclusions

In summary, we have successfully developed an automated HCI assay to quantitate MNGCs induced by Bp in macrophages. This assay was then used to characterize the phenotype of the Bp mutants for their ability to induce MNGC formation and identify small molecules that interfere with this process. This assay can also be applied to identify the molecular targets and mechanisms responsible for the Bp induced phenotype, which to date are poorly understood. In addition, this assay has potential application for characterizing bacterial isolates as well as the identification of immune modulators such as cytokines that induce or inhibit this phenotype. Currently, we are not aware of a robust and direct HCI method to unambiguously distinguish cell clumps from MNGC. Nevertheless, in the experimental conditions described in the manuscript, and in the absence of tested compounds, the detection of MNGC via our HCI method is clearly dependent on infection by Bp (Figures 1, 4 and 5). Compounds that induce cell clumping rather than MNGC-formation might be counter-screened by measuring MNGC formation in mock infected/compound treated cells. In addition, it will be of future interest to develop and implement calculated cellular attributes (such as Cell Area) or the IF staining of additional cellular structures (such as Actin or Tubulin) to further refine and improve the HCI analysis of MNGC.

### Experimental procedures

#### Bacterial propagation

*Burkholderia pseudomallei* K96243 was maintained in either Luria-Bertani (LB) broth, on LB plates or on 1.5% agar plates containing 5% sheep blood (SBA). Broth cultures were grown at 37°C with shaking at 250 rpm and agar plates were incubated at 37°C. For macrophage infections, Bp was grown on LB plates for ~18 h at 37°C and a loopful of the culture was suspended in 10 ml of Dulbecco's Modified Eagle Medium (DMEM) (Gibco, Carlsbad, CA). Bacterial concentrations were determined by measuring the OD_600_ and cell suspensions were adjusted to a multiplicity of infection (MOI) of 30 using a conversion factor of 5 × 10^8^ CFU/ml per unit of optical density at 600 nm [72]. All Bp manipulations were performed in biosafety level 3 laboratories.

#### Construction of a *B. pseudomallei* Δ*bsaZ* type three secretion mutant

Genomic DNA from Bp *Δ*sctUBp3 [70] was purified [73] and used as template DNA for PCR amplification of the Δ*bsaZ* gene. Gene amplification was performed using the forward primer bsaZ-FXb 5’-CATG**TCTAGA**CTTCACGTCACGTCATGCCGAGCGACACG-3’ and reverse primer bsaZ-RH 5’-CATG**AAGCTT**TGTTGGCTAGTGGTCGTTCCC-3’ with the Epicentre FailSafe Kit with buffer “D” (Epicentre Technologies, Madison, WI) using the following conditions: one cycle at 94°C for 5 min; 30 cycles at 94°C for 30 sec, 56°C for 30 sec, and 72°C for 1 min; followed by a final 7 min extension at 72°C. Characters in boldface in the above primer pair represents the XbaI and HindIII sites incorporated into the oligonucleotides for directional cloning. PCR products were resolved on a 2% agarose gel and excised using the GeneClean III kit (Qbiogene, Carlsbad, California). Purified PCR fragments were digested with XbaI and HindIII (New England Biolabs, Ipswich, MA) using buffer “2”, cleaned as described above and cloned into the similarly digested *sacB*-based suicide plasmid pMo130 [74] to generate pMoΔ*bsaZ*. Ligations were transformed into chemically competent *Escherichia coli* TOP10 (Invitrogen, Carlsbad, CA) and recombinant plasmids were purified using the Wizard Plus SV miniprep kit (Promega, Madison, WI). pMoΔ*bsaZ* was electroporated into *E. coli* S17-1 and mobilized into Bp K96243 as previously described [75,76]. pMoΔ*bsaZ* was resolved from transconjugants by culturing the isolates in LB without NaCl containing 10% (wt/vol) sucrose for 3–4 days at 25°C. Deletion of the Bp *bsaZ* gene was confirmed using PCR and apparent by a reduction in the amplicon size of ~1060 bp.

#### Tissue culture and macrophage infections

The RAW264.7 cell line was maintained in DMEM (Gibco) containing 10% FBS (Hyclone, Logan, UT), 1% non-essential amino acids (Sigma, St. Louis, MO), 1% HEPES buffer (Gibco) and 1% L-Glutamine at 37°C under an atmosphere of 5% CO_2_. For macrophage infections, BD Falcon 96-well plates (Franklin Lakes, NJ) were seeded with ~2 × 10^4^ cells/per well and incubated overnight as described above to obtain ~4 × 10^4^ cells/well. Macrophages were infected with Bp at a MOI of 30 (or otherwise noted) for 2 h, monolayers washed three times with PBS to remove extracellular bacteria and either macrophages were fixed (2 h infection) or pre-warmed DMEM containing 10% fetal bovine serum and 250 μg/ml of kanamycin (Sigma) was added to reduce extracellular bacterial growth. Infections were continued for an additional 8 h (or otherwise noted) and monolayers were fixed for ~18-24 h with 10% formalin prior to antibody staining.

#### Macrophage and bacterial staining

Following macrophage fixation cells were washed and subsequently permeabilized for 15 minutes at room temperature with Cellomics 1× permeabilization buffer (Halethorpe, MD), washed twice with PBS and blocked (minimum of 1 h) with Cellomics 1x blocking buffer. Following incubation, blocking buffer was removed and replaced with 50 μL of a 1:1000 dilution of 2 mg/mL anti-*Burkholderia pseudomallei* monoclonal antibody (AB-BURK-P-MAB3, Critical Reagents Program, Frederick, MD) for 1 h. Unbound primary antibody was removed by two washes with PBS and a 1:500 dilution of Dylight 488 goat anti-mouse secondary antibody (Fisher Scientific, Waltham, MA) was added at room temperature for 30 min. Cells were washed two additional times with PBS and 1× CellMask DeepRed (Invitrogen) and 1:10,000 Hoechst nuclear stain (Invitrogen, Carlsbad, CA) were added.

#### Image acquisition and analysis

An Opera QEHS confocal system (PerkinElmer, Waltham, MA) was used for high-throughput image acquisition. 4 imaging fields per well were acquired with a 20X water objective in the Blue (Hoechst 33342), Green (Alexa488) and Far Red (CellMask DeepRed) channels on a single Z-plane in 2 sequential exposures. The first exposure utilized the 488 nm and 640 nm excitation lasers, the emitted light was first filtered by a 405/488/640 primary dichroic mirror, then collected on separate high resolution CCD cameras through 525/35 nm and 690/70 nm band pass filters, respectively. The second exposure used the 405 nm and the excitation light was filtered first through a 405/561/640 primary dichroic mirror, then through a 568 nm Detection dichroic mirror and finally through a 450/50 nm band pass filters. Images were imported into Columbus 2.3 database (PerkinElmer) and analyzed with Acapella 2.7 (PerkinElmer). For the MNGC assay, nuclei were first identified using the Hoechst33342 channel image as input, then the cell edges were determined using the CellMask DeepRed channel image, and bacterial spots were detected using the Alexa 488 channel image. The nuclei detection described above generated a first population of objects (Nuclei), for which cellular attributes were calculated (Cell Area, Number of Foci per Cell). Nuclei objects were then clustered together based on the distance of their nuclear bodies (Measured in pixels). Nuclei objects whose nuclear bodies were within a distance of 0 or 1 pixels, depending on the experiment, were considered as part of a single Cluster object. All the cellular attributes of the Nuclei population were then imported (As sums) into the corresponding Clusters and the number of Nuclei per Cluster attribute was also calculated. Clusters were then further classified into a MNGC subpopulation based on the number of nuclei present in the cluster (Nuclei per Cluster >3). The Percentage of MNGC was calculated as (Number of MNGC objects)/(Number of Cluster objects)*100. Values in the histograms represent the mean +/SD of 6 replicates on the same plate run on 3 separate days (n = 18). Statistical significance for differences in cellular and bacterial attributes between different samples was calculated using the t-test. For single cell analysis presented in Figure 2, images were directly analyzed after image acquisition with Acapella 2.6, (Using an image analysis strategy similar to the one just described above, Nuclear distance for clustering: 3 pixels) and the image analysis results were imported into FCSExpress4 (Denovo Software, Los Angeles, CA), which was used for single cell image cytometry measurements.

#### Small molecule screening in the MNGC assay

RAW264.7 macrophages were seeded as described above. Cells were pre-incubated for 2 h at a final concentration of 20 μM with a collection of 43 compounds selected for their activity on enzymes involved in regulation of chromatin function (Screen-Well Epigenetics Library, version 1.0, Enzo Life Sciences). Cells were then infected with 30 MOI of wild-type Bp K96243. Cells treated with DMSO and infected with Bp K96243 were considered as the negative control; whereas DMSO-treated, mock infected cells were considered as the positive control. Two hours post-infection monolayers were washed three times with PBS to remove extracellular bacteria and pre-warmed DMEM containing 10% fetal bovine serum, the chemical compounds under testing and 250 μg/ml of kanamycin (Sigma) was added to reduce extracellular bacterial growth. Infections were continued for an additional 6 h and monolayers were fixed for ~18-24 h with 10% formalin prior to antibody staining. Cells were IF stained and confocal images were acquired as described above. The MNGC HCI analysis procedure was used to calculate the number of nuclei and the percentage of MNGC. The Z-score for these two cellular attributes was calculated as:

$$Z-{\text{Score}}_{\mathrm{ij}}=\;\frac{\left({\text{Sample}}_{\mathrm{ij}}-\mathrm{\mu N}\right)}{\mathrm{\sigma N}}$$

Where: Z-Score_ij_ = Z-Score for well in Row “i” and Column “j”, % Sample_ij_ = Cellular attribute value for well in Row “i” and Column “j”, μN = Mean of the Cellular attribute for the negative controls on the plate, and σS = Standard Deviation of Cellular attribute for the negative controls on the plate. Compounds that had both Number of Nuclei Z-Score_ij_ > -3 (Cytotoxicity filter) and % MNGC Z-Score_ij_ > 3 (Activity filter) were considered as active compounds.

## Abbreviations

MOI: Multiplicity of infection; HCI: High content imaging; MNGCs: Multinucleated giant cells.

## Competing interests

G. Pegoraro was a PerkinElmer employee.

## Authors’ contributions

GP: designed and developed the image acquisition and analysis procedures; DD constructed the Bp ∆*bsaZ* mutant; BE, DL, JO performed all the experiments, RGP conceived the experimental design and drafted the manuscript; SB, RU and DD provided critical review of the manuscript. All authors contributed to writing the manuscript and read and approved the final version.
